# Type 2 Necrotizing Soft Tissue Infection: A Disastrous Diagnosis Not To Be Missed

**DOI:** 10.7759/cureus.23944

**Published:** 2022-04-08

**Authors:** Ameer Aboud, Farhan Maqbool, Raja Sabbagh, Vaughn Whittaker, Brian Donaldson

**Affiliations:** 1 Department of Surgery, Columbia University College of Physicians and Surgeons, Harlem Hospital Center, New York, USA

**Keywords:** type 2 necrotizing soft tissue infection, type 2 necrotizing fasciitis, streptococcus pyogenes, debridement, necrotizing fasciitis, nsti, necrotizing soft tissue infection

## Abstract

Necrotizing soft tissue infections (NSTIs) are severe, life-threatening forms of infection. Tissues from the epidermis to the deep musculature may be affected. This includes necrotizing forms of cellulitis, myositis, and fasciitis. Delayed diagnosis can lead to widespread tissue loss, limb loss, and mortality, representing a group of infectious surgical emergencies requiring time-sensitive aggressive debridement. This article presents a unique case with a particularly ambiguous and vague presentation of type 2, group A streptococcal NSTI in an intravenous drug abuser. This rapidly spreading infection subjected her to profound morbidity, with loss of all four extremities. Type 2 NSTIs are particularly challenging to diagnose as they often present without classic signs of skin changes, subcutaneous air, and crepitus. They also spread more rapidly and, as such, have a higher morbidity and mortality rate than type 1 NSTIs. We are striving to increase physician awareness of such cases, with the aim of earlier recognition and earlier limb and life-saving interventions.

## Introduction

Necrotizing soft tissue infections (NSTIs) are severe, life-threatening forms of infection. They include necrotizing forms of cellulitis, myositis, and fasciitis. Clinically, there is fulminant tissue destruction, systemic inflammatory signs, and associated high mortality. Timely and accurate diagnosis is paramount for emergency radical surgical debridement to occur in addition to antibiotic therapy. These types of infections and their causative organisms have often been referred to as “the flesh-eating disease,” and “flesh-eating bacteria,” respectively, by non-medical personnel. The classically described form of NSTI, necrotizing fasciitis, is an infection of the deep soft tissue, where there is progressive destruction of the muscle fascia and overlying subcutaneous fat. Infection typically spreads along the muscle fascia due to its relatively poor blood supply, sparing the muscle tissue and dermis because of its abundant blood supply. This results in infection spread along the fascia and subcutaneous tissues, with initial absence of overlying skin findings. This makes necrotizing fasciitis difficult to diagnose without direct visualization of the fascia. The development of sensory deficits may precede the appearance of skin necrosis and provide a clue to the presence of necrotizing fasciitis [1-3].

NSTIs are further classified according to the causative organisms, and the two major groups are type 1, polymicrobial, and type 2, monomicrobial. The classically described crepitus with subcutaneous air on imaging is seen in type 1, which is the most common type of NSTI. However, this article will focus on type 2 NSTIs, which are less common and tend to be more difficult to diagnose due to the absence of crepitus or subcutaneous air on imaging. This, combined with the ability for rapid spread within hours, makes the diagnosis particularly difficult and of utmost consequence to miss.

The diagnosis is a surgical one and is characterized by dishwater-gray exudate, friable superficial fascia, and a notable absence of purulence. The mainstay of treatment is prompt, aggressive surgical debridement to healthy, viable tissues to control the spread of the infection. Any delay in surgical management results in increased tissue loss, limb loss, and higher mortality. Attempts at conservative management of this condition result in a 100% mortality rate. We seek to increase physician awareness of such cases and their variable presentations, with the aim of earlier recognition and earlier limb and life-saving interventions [1-3].

## Case presentation

Our case is of a 48-year-old, African American female with a history of intravenous (IV) heroin abuse and multiple admissions for cellulitis and draining abscesses of the extremities in the past. She presented to the emergency room with 48 hours of body pain, aches, abdominal pain, and weakness. The patient endorsed using IV drugs earlier that day. She complained of severe pain in the right upper extremity.

**Figure 1:**
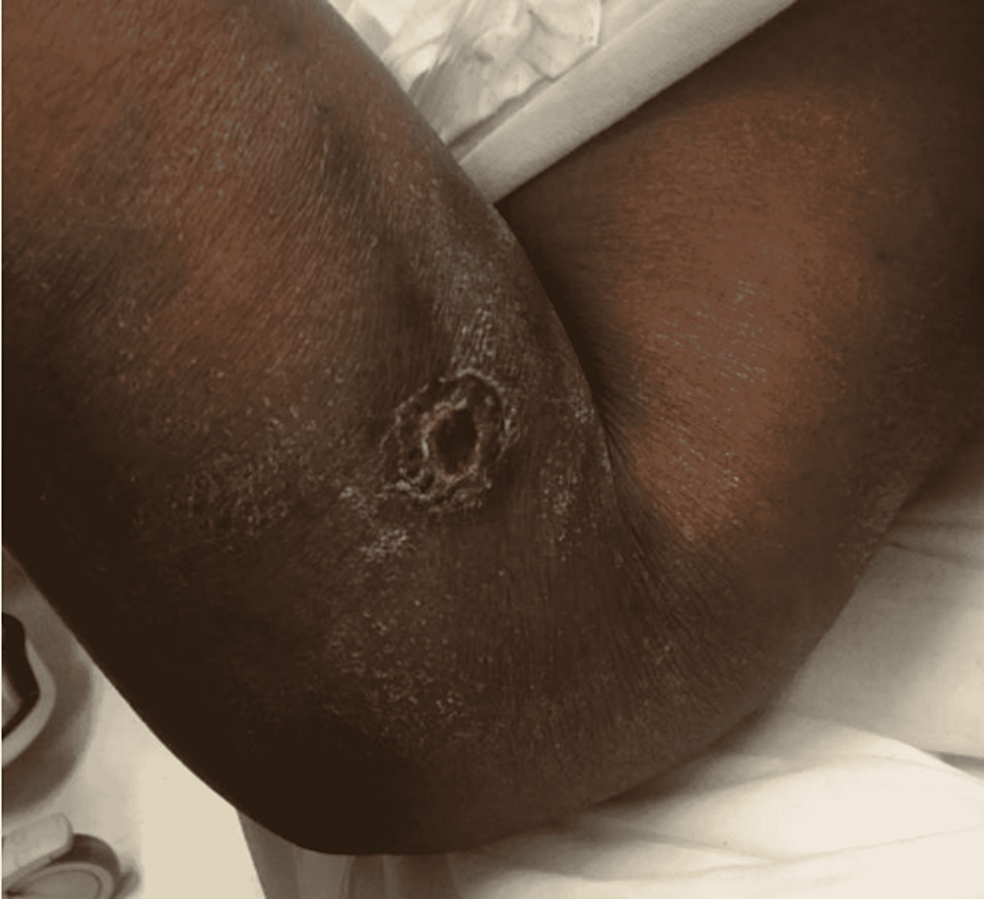
Right forearm self-induced venous puncture ulcer. Initial presentation at 0100 hours, chronic-appearing, self-induced venous puncture ulcer with cellulitis, absent discharge, minimal edema, and minimal to no skin changes.

On examination, the patient was hypotensive to 80/60s mmHg and tachycardic to 120 beats/minute. The examination was also significant for multiple ulcerations on the bilateral upper and lower extremities. Most notably, two ulcers on the right forearm near the antecubital fossa were particularly tender (Figure 1). The right forearm was swollen compared to the left. There was no evidence of crepitus, fluctuance, or drainage. The compartments were soft and the pulses were palpable with a normal sensorimotor examination.

Two sets of blood cultures were obtained. Further, fluid resuscitation with crystalloid and broad-spectrum antibiotics, vancomycin and piperacillin/tazobactam, were initiated immediately.

Laboratory analysis revealed an anion gap metabolic acidosis with a pH of 7.19 and lactate of 6.9 mmol/L. She had an acute kidney injury (AKI) with a blood urea nitrogen (BUN) level of 77 mg/dL and creatinine of 6.5 mg/dL. The white blood cell (WBC) count was 1.67 × 10^3^/µL and the international normalized ratio (INR) was 1.63. She was admitted to the medical intensive care unit (ICU) for septic shock with multiorgan dysfunction.

**Figure 2:**
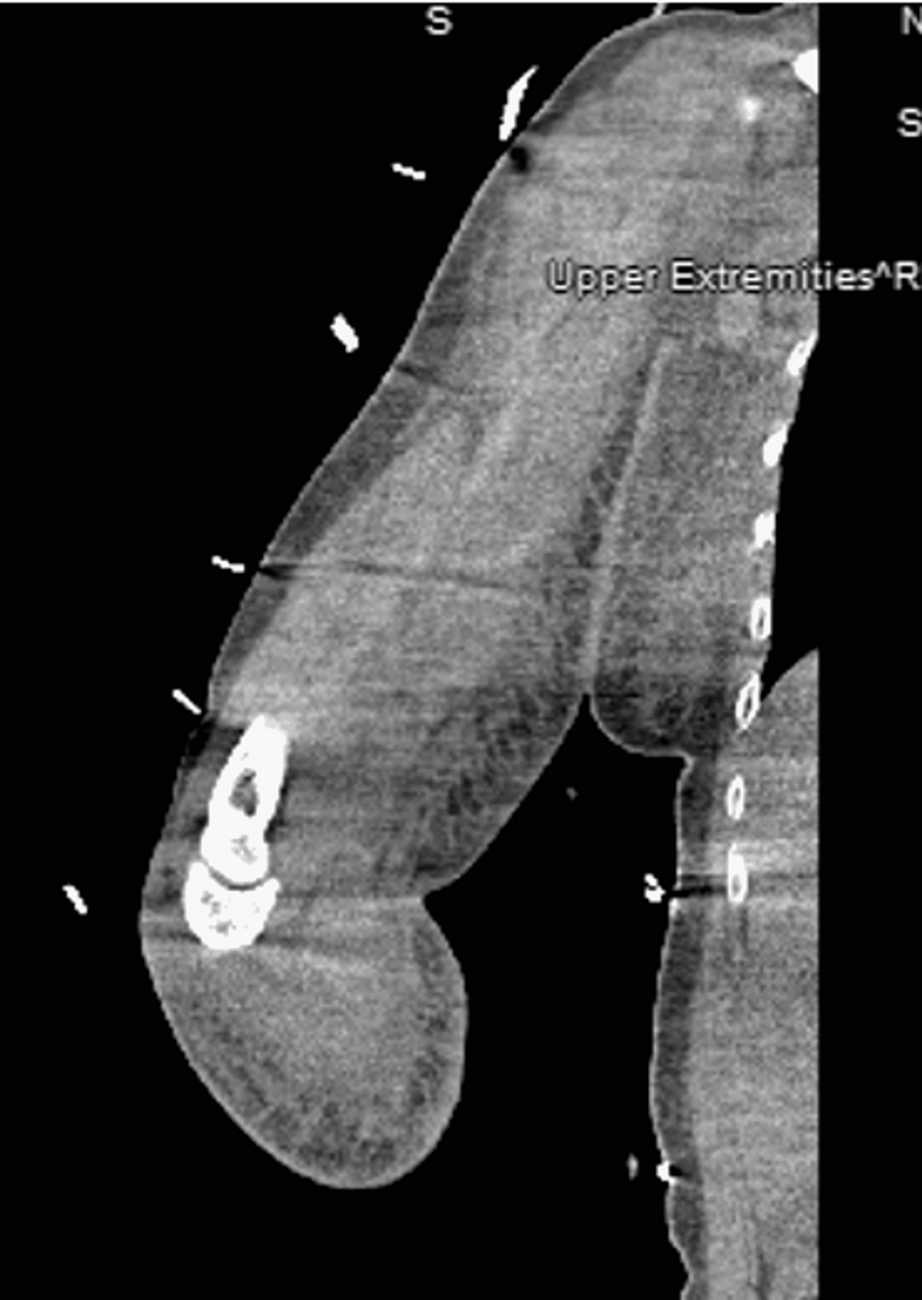
CT without contrast of the right upper extremity. CT with lack of subcutaneous air, nonspecific soft tissue edema, and fat stranding. CT: computed tomography

A surgical consult was obtained; however, due to the unclear nature of the disease and nonspecific symptoms, computed tomography (CT) without contrast of the right upper extremity was ordered. The CT showed nonspecific signs such as diffuse soft tissue swelling, fat stranding, and lymphadenopathy of the right medial upper extremity extending to the right axilla (Figure 2).

**Figure 3:**
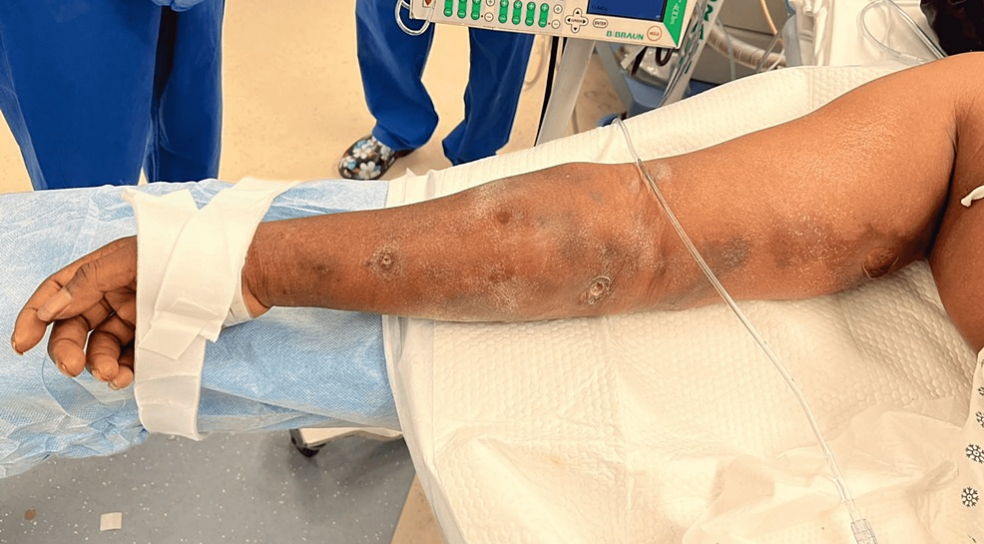
Right upper extremity wound progression. 0600 hours: chronic-appearing ulcers with ecchymosis now extending medially toward the axilla.

On serial examination three hours later, the patient was noted to have a significant increase in pain to the right upper extremity out of proportion to the examination along with acute painful distress. Ecchymosis and purple skin changes had developed in the interval and were extending along the medial aspect of the upper extremity toward the axilla (Figure 3). She had developed a significant motor deficit in this interval, with 0/5 strength of the hand, forearm, and shoulder. She remained hypotensive and tachycardic and unresponsive to volume resuscitation and antibiotics, requiring norepinephrine (20 µg/kg/minute). With this change in the clinical picture, she was taken immediately to the operating room (OR) for radical debridement of the right upper extremity, with concern for an NSTI. An accurate LRINEC score could not be calculated at this time due to the lack of C-reactive protein (CRP) levels.

**Figure 4:**
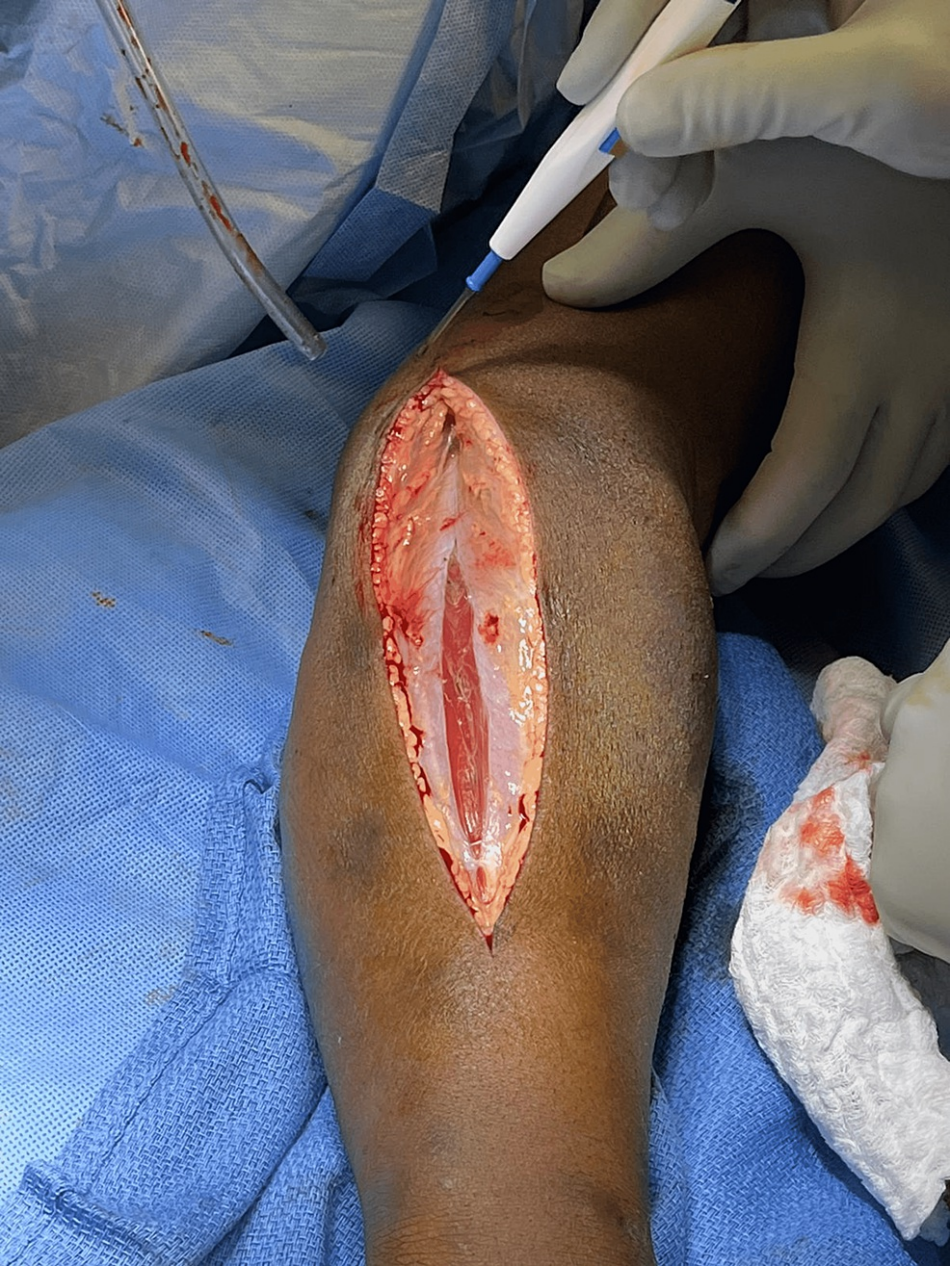
Intraoperative findings. Relatively healthy fascia and muscle with diffuse subcutaneous edema and minimal bleeding. Highlights the possibility of life-threatening necrotizing cellulitis with myofascial sparing.

**Figure 5:**
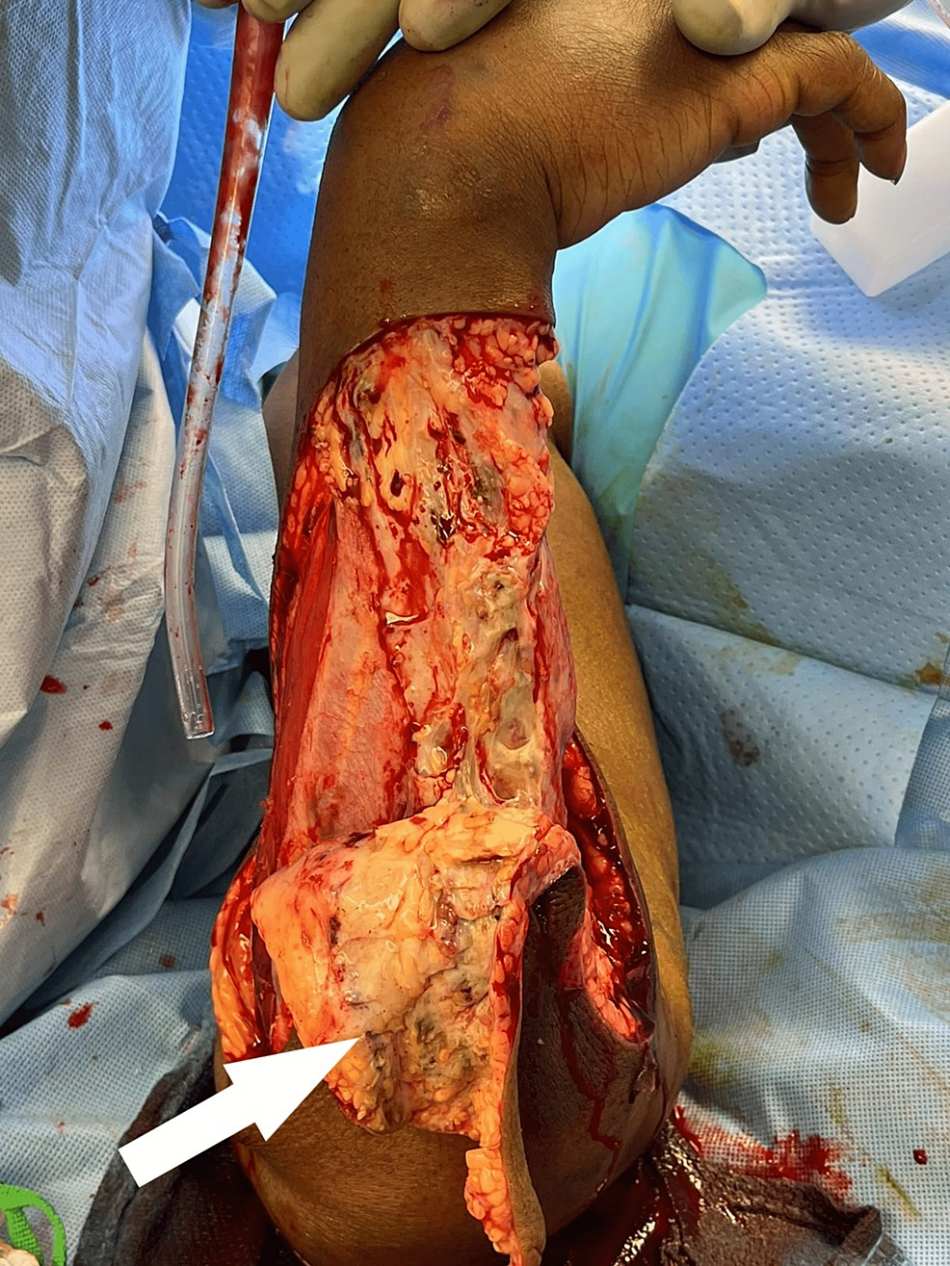
Radical debridement. Minimally bleeding tissues with multiple areas of venous thrombosis (white arrow).

**Figure 6:**
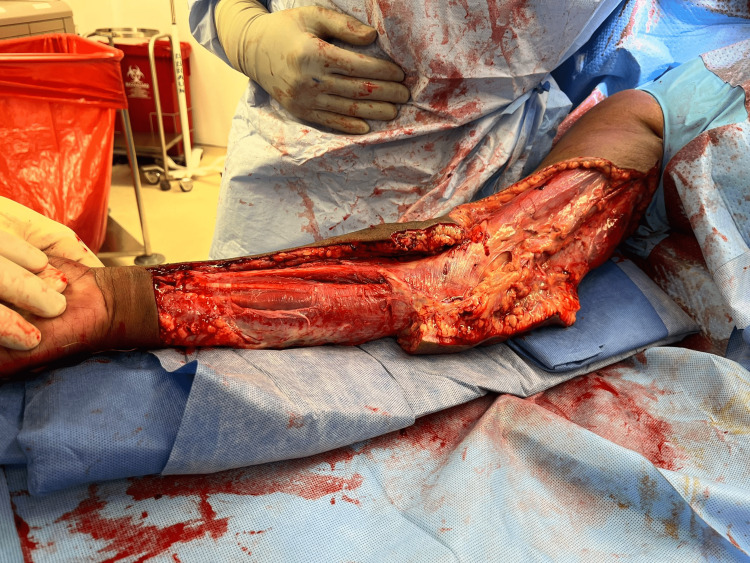
Completed initial radical debridement.

Intraoperatively, the fascia was noted to be grossly healthy appearing, with no frank purulence noted. The findings were subtle, without obvious necrosis. The subcutaneous tissue and fat were very edematous with a slight gray tinge and easily separated with blunt dissection of a finger sweep. The muscles appeared grossly healthy, with edematous, albeit otherwise healthy-appearing fascia (Figure 4). The tissues were not bleeding normally, with multiple areas of venous thrombosis noted, and radical excision of the affected tissue was performed (Figure 5). Proximally, at the medial aspect of the upper arm, similar findings of the fat and subcutaneous tissues were noted and excised. The basilic vein was noted to be thrombosed. Distal pulses were successfully obtained with Doppler throughout the extremity. Fasciotomy of all muscle compartments of the forearm and upper arm was performed. After the initial radical debridement, the remaining tissues appeared healthy and bleeding (Figure 6). The patient was experiencing increasing vasopressor requirements despite two units of packed red blood cell (PRBC) transfusion and crystalloids; therefore, we dressed the wounds with betadine-soaked gauze and transferred her to the surgical intensive care unit (SICU) for further resuscitation.

**Figure 7:**
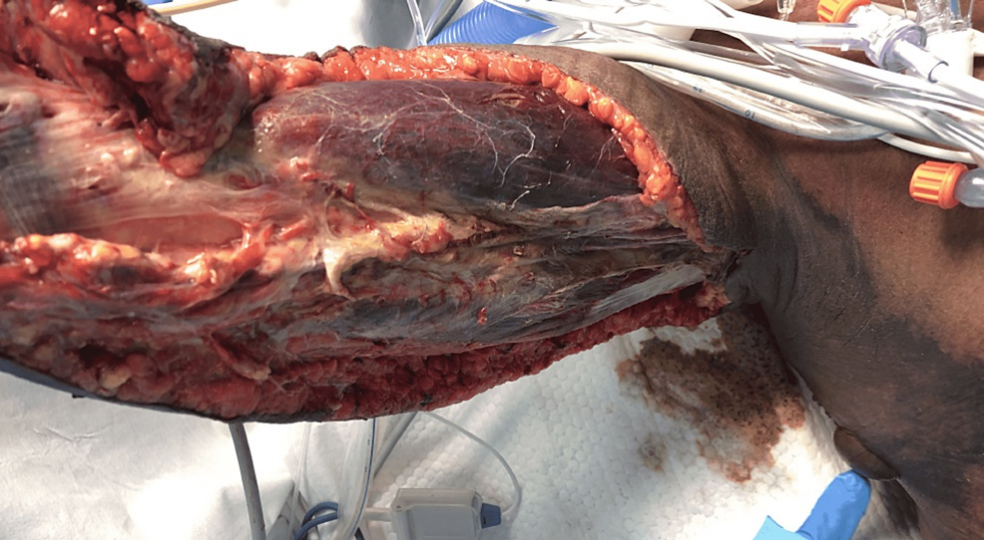
Postoperative day one wound progression. Dusky-appearing muscle and ecchymosis now extending into the axilla and lateral chest wall.

**Figure 8:**
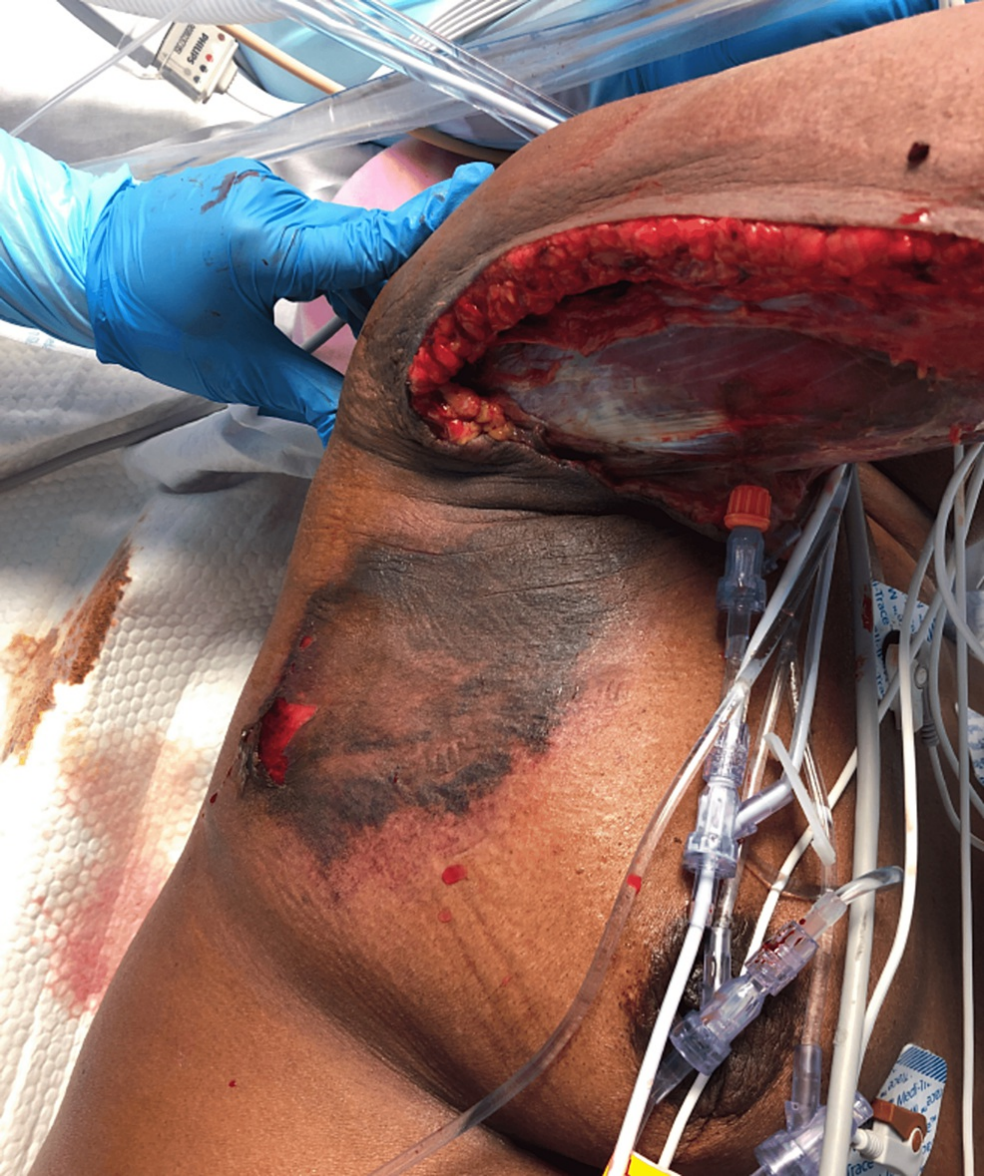
Postoperative day one wound progression. Ecchymosis seen progressing onto the lateral chest wall.

Over the next 16 hours, the patient briefly improved with decreasing pressor requirements and a downtrend of lactate to 3.5 mmol/L. Shortly after, she began to deteriorate again with increased pressor requirements and increased lactate to 5.9 mmol/L. The right upper extremity muscles and the neurovascular bundle was notably dusky and edematous. There were now skin changes extending from the axillary region onto the lateral chest wall (Figures 7, 8).

**Figure 9:**
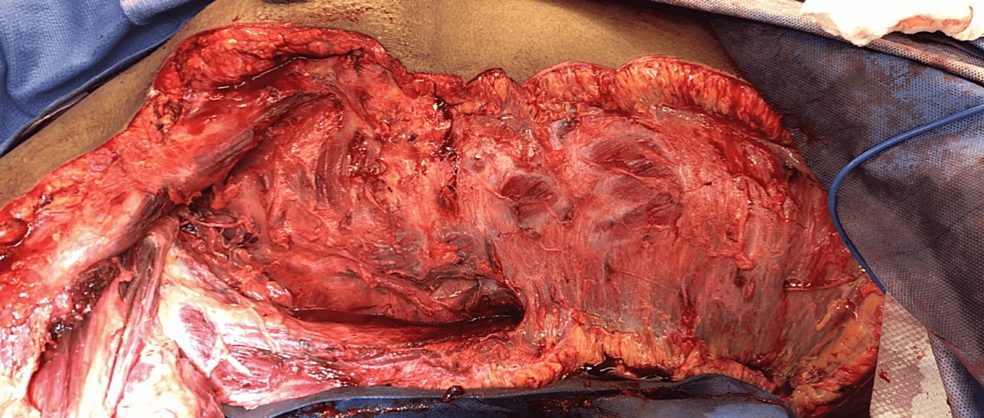
Radical debridement of the lateral torso wall. Second radical debridement (just prior to right shoulder disarticulation).

She was immediately returned to the OR for further debridement. The shoulder skin and subcutaneous tissues were debrided and incisions extended to the lateral chest wall. The tissues were nonviable nonbleeding, and, subsequently, the lateral torso soft tissues were excised to the level of the iliac crest. Only at this point were the tissues firmly adherent, healthy-appearing, and bleeding. The rapid spread of the infection with subsequent involvement of the axilla and neurovascular bundle was thought to have compromised the entire right arm. The arm was frankly necrotic with multiple areas of venous thrombosis. The family was informed of the necessity for amputation as a life-saving procedure, and she agreed. A right shoulder disarticulation was performed (Figure 9).

The left forearm was explored with two incisions, volar and dorsal, to explore ecchymosis that had developed in the interim, ruling out an additional source of infection. The tissues were noted to be healthy and bleeding. A diagnostic laparoscopy was performed to rule out any intraabdominal source as a CT abdomen/pelvis performed on admission showed nonspecific dilated loops of bowel with a possible transition point. There was no significant pathology or mechanical obstruction noted upon diagnostic laparoscopy, attributed to likely ileus. She was returned to the SICU for continued resuscitation. The pH, lactate, and vasopressor requirements began to improve. Blood cultures and surgical swab cultures returned positive for group A, beta-hemolytic *S. pyogenes*, indicative of a type 2 NSTI.

On postoperative days (PODs) three to five, daily dressing changes continued with bedside debridement of nonviable tissues. She developed symmetric peripheral dry gangrene of all three remaining extremities. She began to bleed from the wound, IV sites, and from the nares. The platelets began to downtrend to a nadir of 2 x 10^3^/µL from 220 x 10^3^/µL, hemoglobin trended from 10.4 g/dL to 6.3 g/dL. The INR rose to 2.8. She experienced another deterioration in vasopressor requirements. A diagnosis of disseminated intravascular coagulation (DIC) was made, with likely microvascular thrombosis resulting in the symmetric peripheral gangrene. She received six units of PRBC, two packs of pooled platelets, and four units of fresh frozen plasma (FFP).

**Figure 10:**
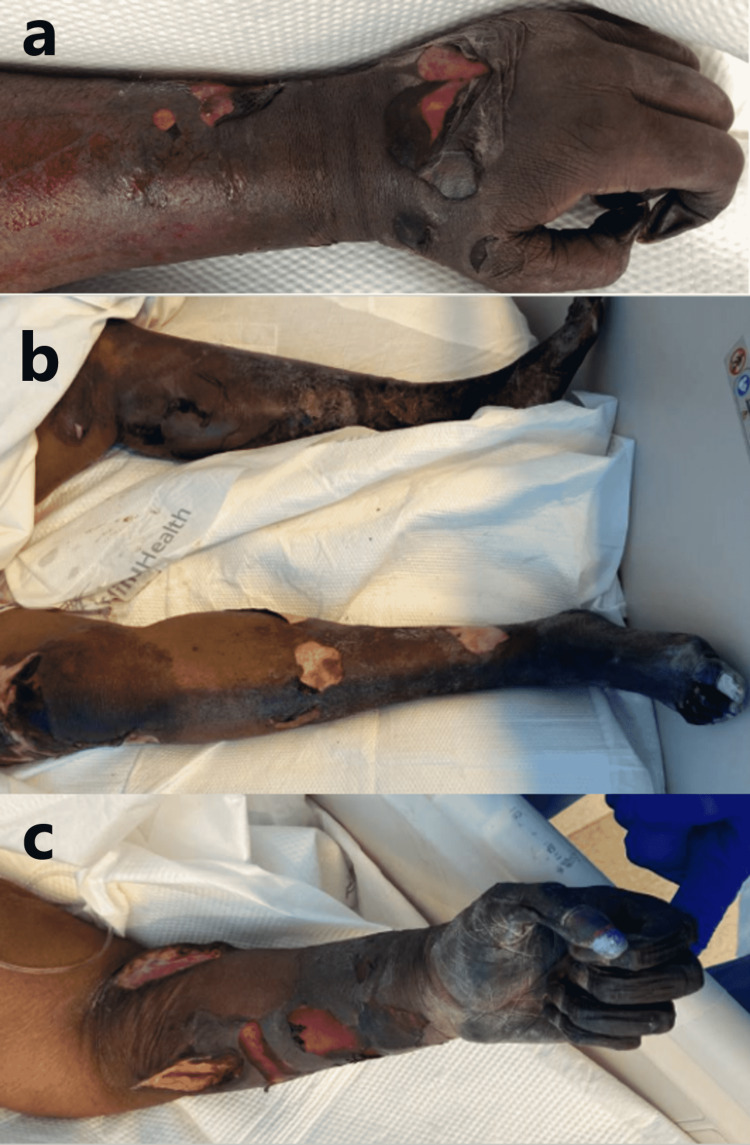
Symmetrical peripheral gangrene. (a) POD five: dusky, cold-appearing extremities with epidermal sloughing. (b, c) POD 12: demarcation of gangrenous changes. POD: postoperative day

On PODs five and six, the patient improved and was weaned off of pressor support. The symmetric peripheral gangrene continued to demarcate (Figure 10).

**Figure 11:**
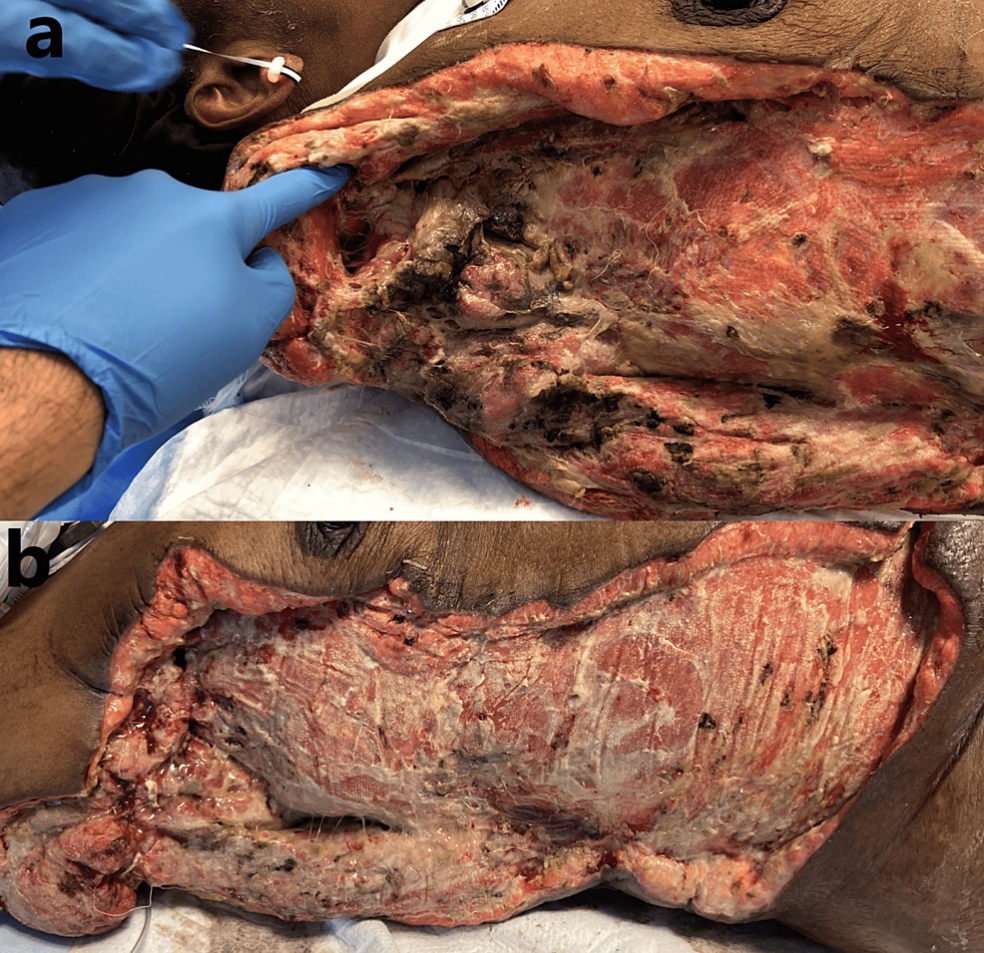
Status post-shoulder disarticulation with the necrotic neurovascular bundle. (a) POD 14: nonviable tissue involving the axillary neurovascular bundle. (b) Status post-debridement and coverage with deltoid to pectoralis/latissimus muscle flap. POD: postoperative day

On POD 14, the patient was taken for re-exploration with sharp debridement. There were necrotic skin changes that had developed in the axilla surrounding the neurovascular bundle. The axillary artery was isolated and all surrounding necrotic tissue was debrided. The axillary artery, vein, and nerve were isolated and ligated appropriately, with debridement of the distal questionable ends. The neurovascular bundle was then covered with deltoid-to-pectoralis major and deltoid-to-latissimus dorsi muscle flap (Figure 11).

**Figure 12:**
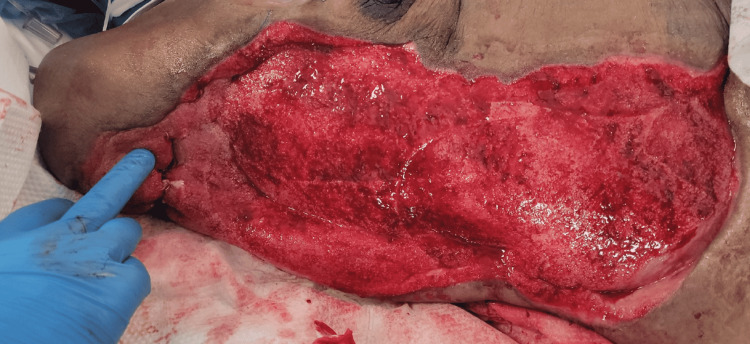
Status post-negative pressure wound therapy. Healthy, abundant, beefy red granulation tissue post-application of negative pressure wound therapy device.

A tracheostomy was created. She tolerated continuous positive airway pressure (CPAP) and trach collar. An oral diet was started after clearance from Speech and Swallow service. Local wound care continued at the bedside, and, eventually, a wound vac was placed with subsequent healthy granulation of tissue throughout the entire wound bed (Figure 12).

With the wound bed now healthy with an abundance of granulation tissue, a pedicled latissimus dorsi muscle flap is planned by the plastic surgery service for coverage, as well as split-thickness skin grafting.

Regarding the patient’s three remaining limbs, final demarcation revealed complete dry gangrene of the wrist, hand, and bilateral feet below the ankles. Due to the noninfectious nature of the dry gangrene, elective below the elbow, and bilateral below-knee amputations will be required after her primary wound is healed.

## Discussion

NSTIs are uncommon but severe forms of infection with high associated morbidity and mortality. The diagnosis is wrought with pitfalls, and a high index of suspicion should be maintained, as well as a thorough understanding of their etiology and pathogenesis. Awareness of the common pitfalls associated with the diagnosis will shorten the time to diagnosis and surgical treatment. Patients may not present febrile due to nonsteroidal anti-inflammatory drugs or other antipyretics taken to relieve their pain prior to presentation. There may be an absence of any cutaneous manifestations as the infection may be cryptogenic or via hematogenous spread. Severe pain out of proportion to the examination may be erroneously attributed to traumatic injury or a recent surgical procedure. Radiographic imaging may show only nonspecific soft tissue edema without subcutaneous gas, which is consistent with noninfectious causes (e.g., trauma, inflammation). And finally, the high prevalence of systemic manifestations of NSTI such as nausea, vomiting, diarrhea, and abdominal pain may confound the diagnosis, causing further delay [1].

NSTI may begin from inoculation of bacteria via any break in the skin, including seemingly minor wounds, surgical wounds, or even intact skin. The complete absence of any skin breakdown should also not completely rule out an NSTI. There is evidence to suggest that 50% of type 2 NSTIs are caused by hematogenous spread via minor muscular contusion or strain which leads to injury of the myocytes. These injured myocytes, in the setting of transient bacteremia from the nasopharynx for example, are more prone to inoculation than healthy myocytes. This allows the development of necrotizing myositis and fasciitis to ensue through a hematogenous route with intact, normal skin. [1] In our patient, as seen in Figure 1, the infection appears to have stemmed from a chronic-appearing ulcer at an IV puncture site. Initially, this was indistinguishable from a usual case of cellulitis. The worrisome features that developed rapidly were the pain out of proportion to examination and the initiation of neurologic deficits.

The two major types of NSTI are based on the causative organism. Type 1, Polymicrobial (70-80%) [4,5]: most common etiology for necrotizing infection. Caused by aerobic and anaerobic bacteria, commonly associated with diabetes, obesity, peripheral artery disease, and other underlying comorbidities. This type of infection may commonly present with subcutaneous air and/or crepitus due to the presence of gas-producing organisms among others [1].

Type 2, Monomicrobial (20-30%) [4,5]: the typical organism is group A *Streptococcus *(GAS) and other beta-hemolytic streptococci. It may occur in any age group and in individuals with no underlying morbidities. This type of NSTI characteristically does not have subcutaneous air/crepitus, leading to an easily missed diagnosis. Type 2 NSTI have a higher mortality rate (>30%) and progress at a notably more rapid rate than type 1 infections. This compounds the consequence of a missed or delayed diagnosis [1-3].

Clinical picture

NSTIs may involve the epidermis, dermis, subcutaneous tissue, fascia, and muscle. The lower extremities are more commonly involved than the upper extremities, particularly in diabetics or those with peripheral vascular disease. These infections most commonly present acutely, but rarely they present sub-acutely, progressing over days. Type 1 NSTIs are more likely than type 2 NSTIs to present sub-acutely, with type 2 NSTIs causing significantly more systemic toxicity and more rapid spread. Rapid progression to extensive tissue destruction may occur, leading to sepsis, septic shock, limb loss, and/or death.

The clinical manifestations are variable, and any combination of the following may be present: erythema without sharp margins (72%), edema extending beyond the visible erythema (75%), severe pain out of proportion to the examination (72%), crepitus (50%), and skin changes such as bullae, necrosis, or ecchymosis (38%). Systemic inflammatory response syndrome including fever and tachycardia is present in 60% of cases. Pain out of proportion to the examination is the most consistent finding identified on physical examination in the presence of an NSTI [3].

In the setting of necrotizing fasciitis, sensory deficits may develop in the involved area due to thrombosis of small blood vessels and destruction of superficial nerves in the subcutaneous tissue. This may precede the appearance of skin necrosis. Subcutaneous gas is often present in the polymicrobial, type 1, form of necrotizing fasciitis, particularly in patients with diabetes [1-3].

Evaluation and initial management

NSTIs are recognized based on the clinical examination, with laboratory analysis providing adjunctive information to support the diagnosis. Not all cases present in a typical fashion, and many present with swelling and erythema alone. Pain that is out of proportion to the examination is the single most consistent finding. Rapid and accurate diagnosis is paramount to improving outcomes and minimizing tissue loss. It is important to maintain a high index of suspicion for NSTIs as the definitive diagnosis is made upon surgical exploration of the soft tissues in the OR.

Based on the timeline when the patient presents, systemic illness and altered mentation may be the only findings. In these cases, a thorough physical examination to expose an occult infection is irreplaceable when a history cannot be elicited. A very high index of suspicion should be maintained for any patient with a soft tissue infection who rapidly deteriorates with organ system failure [1-3]. In our case, the patient initially presented with vague systemic symptoms, signs of systemic toxicity, pain out of proportion to the examination, and with minimal skin findings.

Radiographic imaging can be useful in the event that the examination is limited, such as in cases of intoxication, sedation, morbid obesity, or indeterminant examination. However, if the examination reveals crepitus or rapid progression of clinical manifestations, then surgical intervention should not be delayed for imaging. The most useful radiographic finding is the presence of gas in soft tissues; CT is the most sensitive modality to detect this finding. Although not the most sensitive, plain X-ray films can also reveal subcutaneous air and can be performed more rapidly than CT, or if CT is unavailable [1-3].

Surgical exploration is the gold standard for the diagnosis and treatment of NSTIs. While awaiting OR preparation or in some cases, radiography, a standard septic workup, and empiric broad-spectrum antibiotics should be initiated. Two sets of blood cultures along with a complete blood count, basic metabolic panel, and CRP should be drawn prior to antibiotic administration. IV fluid resuscitation should also be initiated prior to OR exploration as these patients may experience significant fluid shifts and third spacing due to the extensive inflammation, and subsequent wide surgical debridement.

The blood analyses obtained may be used to calculate the LRINEC score (Laboratory Risk Indicator for Necrotizing Fasciitis), which is a useful adjunct in the workup of NSTI. The WBC count, hemoglobin, sodium, glucose, serum creatinine, and CRP levels are used in the calculation of this score. Although studies report the score to have a 92% positive predictive value and 96% negative predictive value, it should never be used in isolation to rule out an NSTI [6]. High suspicion based on the clinical examination is the best indicator for operative exploration.

Upon surgical exploration, intraoperative findings include extensive soft tissue destruction with fascia that is loosely or not adherent to adjoining layers. The skin, subcutaneous tissue, fascial planes, and muscle should be examined for viability. Involved tissue planes are easily separated with blunt dissection of the finger. The soft tissue involvement often extends beyond margins based solely on external appearance. Thrombosis of the underlying dermal capillary network is apparent and precedes overt skin necrosis. Incision through involved areas will often not bleed normally [1-3]. Even with open surgical visualization of the fascia in our patient, the diagnosis was somewhat questioned. The relatively healthy fascia and muscle with a lack of obvious necrotic tissue was unusual, and the possibility that we were faced with just a high-grade cellulitis was contemplated. The ominous finding of tissues that were minimally bleeding and planes easily separated with blunt finger sweep is what forced continued debridement of relatively normal-appearing tissues. This highlights the possibility of life-threatening necrotizing cellulitis with myofascial sparing. As such, even with a radical debridement of the majority of soft tissue from the upper extremity, it is probable that the infection had spread beyond our surgical borders at the time, and it was not yet grossly apparent. This allowed for continued rapid spread onto the thorax and right flank.

Early debridement is associated with decreased morbidity and mortality; survival is significantly increased among patients taken to surgery within 24 hours of admission compared to those in whom surgery was delayed. There is data to suggest that survival is further increased if debridement occurs within six hours. The goal of operative management is to perform wide, aggressive debridement of all necrotic tissue until healthy, viable (bleeding) tissue is reached. It is common for these patients to require serial debridement in the OR every one to two days until the necrotic tissue is no longer present. However, the goal should be to remove all grossly necrotic tissues from the index operation. Upon serial examination, tissues that were not grossly necrotic initially may have demarcated and should then be debrided as well. In severe cases of NSTI involving the extremities, amputation may be needed for adequate source control [1-3].

Once source control is obtained and the infection is brought under control, the wound healing process may begin. There are various methods for wound management after wide debridement. These include skin grafting, various types of soft tissue or muscle flaps, negative-pressure wound therapy, and healing by secondary intention. The extent and anatomical location of the wound would dictate the best option for coverage. An early multidisciplinary approach is optimal, with certain cases requiring general surgery, plastics, vascular, orthopedics, and urology services to be involved.

NSTIs have the potential to be severely debilitating. The mental health impacts cannot be understated. Extremity amputations, especially dominant upper limb loss, represents a high risk for major depression, posttraumatic stress disorder, and psychological distress [7]. Moreover, in our case, functional four extremity limb loss will undoubtedly lead to major psychological consequences and poor quality of life. The socioeconomic effects are also great, with our patient unlikely to be able to return to the workforce, likely leading to permanent disability and complete dependence for activities of daily living. Management of NSTIs may also lead to profound ethical dilemmas, consideration for the psychological and quality of life disturbances that can emerge from these cases demands early, and clear communication to the patient and family about possible prognosis. Determination of the patients’ and families’ wishes early and establishing goals of care is important as patients may easily lose decision-making capacity if critical illness develops or mechanical ventilation is needed.

## Conclusions

Our case was particularly ambiguous, presenting as initial cellulitis around a prior self-induced venous puncture site. Even intraoperative findings were particularly vague, where the diagnosis was questioned due to the relatively healthy-appearing muscle and fascia. A key learning point is that even with healthy-appearing muscle and fascia, necrotizing cellulitis can spread solely within the subcutaneous tissue and reap massive destruction.

With our case report, we sought to increase awareness among physicians regarding the variable presentations and courses NSTIs may take. It is important to remember that they commonly present with nonspecific constitutional symptoms and seemingly minor to no apparent skin changes at the early stage. Once skin findings such as bullae or ecchymosis appear, the underlying necrosis is significant. Patients may complain of severe pain in areas of normal-appearing skin as the infection is spreading, hidden, and along the fascial planes. Laboratory parameters (including LRINEC score) may support but do not rule out the presence of an NSTI. Radiologic findings may be nonspecific and also cannot rule out NSTIs, especially type 2. A delay of only hours not only represents a significant increase in morbidity and mortality but also the associated psychological, ethical, and socioeconomic disastrous consequences. An experienced surgical consultation should be obtained for any question of NSTI and to undergo immediate operative debridement at the mere clinical suspicion for such a diagnosis.

## References

[1] Stevens DL, Bryant AE (2017). Necrotizing soft-tissue infections. N Engl J Med.

[2] Bonne SL, Kadri SS (2017). Evaluation and management of necrotizing soft tissue infections. Infect Dis Clin North Am.

[3] Stevens DL, Baddour LM (2021). Necrotizing soft tissue infections. UpToDate.

[4] van Stigt SF, de Vries J, Bijker JB, Mollen RM, Hekma EJ, Lemson SM, Tan EC (2016). Review of 58 patients with necrotizing fasciitis in the Netherlands. World J Emerg Surg.

[5] McHenry CR, Piotrowski JJ, Petrinic D, Malangoni MA (1995). Determinants of mortality for necrotizing soft-tissue infections. Ann Surg.

[6] Wong CH, Khin LW, Heng KS, Tan KC, Low CO (2004). The LRINEC (Laboratory Risk Indicator for Necrotizing Fasciitis) score: a tool for distinguishing necrotizing fasciitis from other soft tissue infections. Crit Care Med.

[7] Armstrong TW, Williamson ML, Elliott TR, Jackson WT, Kearns NT, Ryan T (2019). Psychological distress among persons with upper extremity limb loss. Br J Health Psychol.

